# Wide limits of agreement between left ventricular stroke volume, right ventricular stroke volume and aortic and pulmonary forward flow

**DOI:** 10.1186/1532-429X-13-S1-P316

**Published:** 2011-02-02

**Authors:** Christian L Polte, Kerstin Lagerstrand, Carl Lamm, Odd Bech-Hanssen

**Affiliations:** 1 Department of Cardiology, Sahlgrenska University Hospital, Gothenburg, Sweden; 2 Department of Radiology, Sahlgrenska University Hospital, Gothenburg, Sweden

## Objective

The aim of the study was to assess the reliability of using left ventricular stroke volume (LVSV), right ventricular stroke volume (RVSV), aortic forward flow (AFF) and pulmonary forward flow (PFF) in the evaluation of valvular regurgitation by studying the extent of agreement between the different parameters in healthy subjects with only trivial valvular regurgitation.

## Background

The quantification of valvular regurgitation by CMR is usually a second-line diagnostic tool that is used when echocardiography has proven inconclusive. According to guidelines are aortic and mitral regurgitations considered severe if the regurgitation volume > 60 ml and regurgitation fraction > 50 %. Regurgitation of a single heart valve may be calculated by comparing LVSV and RVSV derived from multislice ventricular planimetry. In isolated aortic and pulmonary regurgitation, phase contrast velocity sequences can be used as an alternative or complementary method to quantify the regurgitation volume and fraction. This method can be subject to error due to motion of the valve annulus, elastic expansion of the aortic root and inherent sequence errors. In the case of isolated mitral and tricuspid regurgitation the LVSV or RVSV can alternatively be set in relation to flow measurements in the aorta and pulmonary artery for grading the regurgitation.

## Methods

Twenty healthy subjects (10 female and 10 male) underwent CMR imaging (Philips Achieva 1.5T) using steady-state free precession sequences to obtain short axis data sets and phase contrast velocity sequences to obtain aortic and pulmonary flow measurements. All images were analysed repeatedly (ViewForum, Philips) to acquire LVSV, RVSV, AFF and PFF. The agreement between the LVSV and AFF, PFF and RVSV was calculated using Bland-Altman analysis. The grading of valvular regurgitation was determined using echocardiography.

## Results

**Figure 1:**
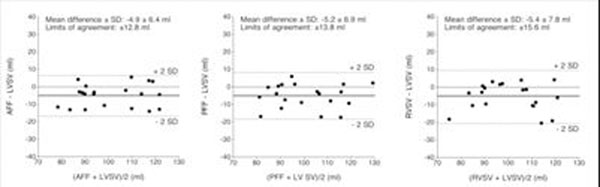
Bland-Altman diagrams demonstrating the mean difference (solid lines) and limits of agreement (dashed lines) between AFF and LVSV (left), PFF and LVSV (middle) and RVSV and LVSV (right).

Trivial regurgitation was found in the mitral (n=9), pulmonary (n=14) and tricuspid (n=19) valves. The mean difference of the LVSV was significantly larger compared with that of the AFF (p=0.003), PFF (p=0.003) and RVSV (p=0.006) (Figure 1). According to the limits of agreement (2 SD), can the calculated regurgitation volume vary in the range of ± 13 to 16 ml.

## Conclusion

The limits of agreement when comparing LVSV with AFF, PFF and RVSV were relatively wide. In clinical practice this might introduce a significant error when calculating the regurgitation volume and fraction. From our results it can be questioned if it is advisable to base the grading of valvular regurgitation severity solely on these two parameters.

